# Multiplex long-amplicon sequencing for comprehensive molecular surveillance of *Plasmodium falciparum* resistance to artemisinin and partner drugs in artemisinin-based combination therapies (ACTs)

**DOI:** 10.1186/s13071-025-07058-6

**Published:** 2025-10-21

**Authors:** Fei Li, Jiayao Zhang, Sui Xu, Yinlong Wang, Zhiyi Mao, Guoding Zhu, Yaobao Liu, Jun Cao

**Affiliations:** 1 School of Public Health, Nanjing Medical University, Nanjing, Jiangsu 211166 People’s Republic of China; 2 National Health Commission Key Laboratory of Parasitic Disease Control and Prevention, Jiangsu Provincial Key Laboratory On Parasite and Vector Control Technology, Jiangsu Provincial Medical Key Laboratory, Jiangsu Institute of Parasitic Diseases, Wuxi, Jiangsu 214064 People’s Republic of China

**Keywords:** *Plasmodium falciparum*, Long-amplicon sequencing, Molecular surveillance, Drug resistance

## Abstract

**Background:**

Antimalarial resistance, especially artemisinin resistance, in *Plasmodium falciparum* threatens global malaria control. Molecular surveillance of *Plasmodium falciparum* drug resistance is critical for monitoring the efficacy of artemisinin-based combination therapies (ACTs). Current molecular surveillance tools for *Plasmodium falciparum* artemisinin resistance are restricted to predefined polymorphism hotspots in limited loci (*Pfk13* domain), failing to capture novel mutations in emerging resistance genes (*Pfcoronin*, *Pfubp1*, *Pfap2μ*) or cover complete coding regions. This study addresses this gap by developing an optimized long-amplicon panel for comprehensive, full-gene resistance surveillance.

**Methods:**

Six genes were selected for the multiplex PCR amplification, including four artemisinin resistance-related markers (*Pfk13*, *Pfcoronin*, *Pfap2μ* and *Pfubp1*) and two ACTs partner drugs resistance markers (*Pfmdr1* and *Pfcrt*). Amplicons were standardized to 2.5 ± 0.2 kb using *multiply* software to minimize amplification bias. The full-length coverage of *Pfk13*, *Pfcoronin* and *Pfap2μ* was achieved. Primer concentrations and annealing temperatures were iteratively optimized through gel electrophoresis and sequencing validation. Analytical validation was assessed using 11 mock samples (parasitemia ranging from 1% to 0.0001%) and 16 field-collected venous blood (VB) samples. The samples were processed via Illumina paired-end sequencing.

**Results:**

The long-amplicon panel was specifically designed to cover the full-length coding region of the *Pfk13* gene and co-amplify three artemisinin resistance-related molecular markers. It can exhibited species-specific amplification efficiency for *Plasmodium falciparum* targets with undetectable cross-reactivity against non-falciparum *Plasmodium* species. Analytical sensitivity thresholds were at 50 parasites/μL for dried blood spots (DBS) sample and 5 parasites/μL for VB sample, with all targets achieving 100% coverage. Notably, DBS samples over 50 p/μL required only 0.25GB of sequencing data (mean depth: 55 ×) for complete target coverage, while VB samples above 5 parasites /μL required 0.5GB data (mean depth: 33 ×) still keeping over 89% coverage uniformity. The total cost per sample was minimized to $15.60, encompassing PCR amplification, library preparation and sequencing.

**Conclusions:**

The new long-amplicon panel improves malaria molecular drug-resistance monitoring by detecting known and emerging mutations across entire genes in a single test. The high sensitivity for ultralow parasitemia samples, coupled with their species specificity and cost-effectiveness, makes it a scalable tool for monitoring multidrug-resistant *P*. *falciparum* strains, particularly in resource-limited settings.

**Graphical Abstract:**

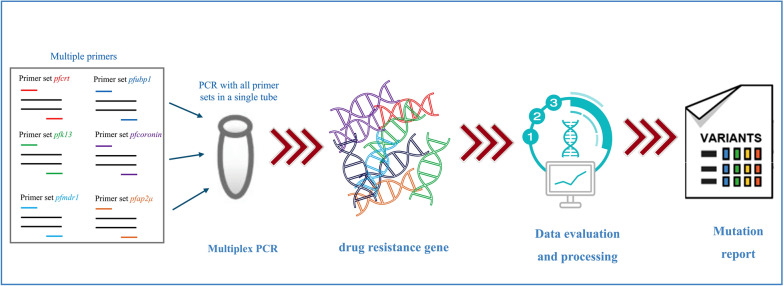

**Supplementary Information:**

The online version contains supplementary material available at 10.1186/s13071-025-07058-6.

## Background

Malaria remains a life-threatening infectious disease with profound implications for global health. The World Health Organization (WHO) reported approximately 263 million malaria cases and 597,000 deaths globally in 2023, with Africa region accounting for 94% of cases and 95% of deaths [1]. At present, the primary treatment for *Plasmodium falciparum* malaria, the most virulent form of malaria, is artemisinin-based combination therapies (ACTs). These combine a fast-acting artemisinin derivative with a longer-lasting partner drug to ensure effective parasite clearance and lower the risk of recrudescence [2]. The most widely used ACTs are artemether-lumefantrine (AL), artesunate-amodiaquine (AS-AQ), artesunate methylfluoroquine (AS-MQ) and dihydroartemisinin-piperaquine (DHA-PPQ) [3]. However, the widespread deployment of ACTs has driven the evolution of artemisinin resistance (ART-R) and partner drugs resistance in *Plasmodium falciparum*. Currently, artemisinin-resistant *Plasmodium* strains have spread across the Greater Mekong Subregion (GMS) [4, 5]. Independently originated artemisinin-resistant parasite strains have been reported in Africa, South America, and Oceania [6–9]. In addition, multiple investigations have documented the parasite strains resistant to both artemisinin and partner drugs in Southeast Asia and Africa [10, 11]. In Southeast Asia, artemisinin and piperaquine-resistant have been identified through longitudinal surveillance [12]. Concurrently, declining sensitivity to both artemisinin and lumefantrine has been observed in northern Uganda [13].

Functional genomic studies and population-level analyses have elucidated key resistance mechanisms, including genetic variants linked to artemisinin delayed parasite clearance and reduced susceptibility to ACTs partner drugs [14–16]. Mutations in the Kelch13 propeller domain (*Pfk13*; PF3D7_1343700) on chromosome 13 are most strongly associated with delayed parasite clearance under artemisinin pressure [14]. However, these mutations do not account for all artemisinin resistance cases [17]. For example, mutations in the *P*. *falciparum* coronin (*Pfcoronin*; PF3D7_1251200 )[18]*, P. falciparum* ubiquitin carboxyl-terminal hydrolase 1 (*Pfubp1*; PF3D7_0104300) [19] and *P*. *falciparum* AP-2 complex subunit mu (*Pfap2μ*; PF3D7_1218300) [20] are also linked to ART-R and could become new ART-R markers. A study on imported African ACTs treatment recrudescence cases found no *Pfk13* gene variations but mutations in the *Pfubp1* and *Pfap2μ* genes [21]. Another in vitro study suggested that *Pfcoronin* and *Pfk13* mutations impact ART-R independently [22]. ACTs resistance also involves many molecular markers of partner drugs resistance. *P*. *falciparum* multidrug resistance protein 1 (*Pfmdr1*; PF3D7_0523000) mediates lumefantrine [23] and mefloquine resistance [24]. *P*. *falciparum* chloroquine resistance transporter (*Pfcrt*; PF3D7_0709000) drives piperaquine[25] resistance and contributes to amodiaquine [26] and lumefantrine [27] resistance through distinct mutations. Systematic surveillance resistance-related mutations across endemic regions are critical for tracking epidemiological trends and informing targeted malaria control strategies.

Several amplicon sequencing platforms have been developed for molecular surveillance of *P*. *falciparum* antimalarial drug resistance. Molecular inversion probes (MIPs) efficiently interrogate known resistance markers [28], yet its utility is limited by specific probe design due to complex AT-rich *P*. *falciparum* genome, length of efficiently targetable sequences as well as the diminished sensitivity for detecting low-frequency variants. Recent nanopore-based multiplex PCR [29] and MADHATTER amplicon panels [30] have expanded surveillance capacity for antimalarial drugs resistance, but detection of emerging markers (*Pfcoronin*, *Pfubp1* and *Pfap2μ*) still requires further optimization. These technical constraints show the urgent need for comprehensive profiling tools that can capture both known and emerging resistance mechanisms across complete gene architectures.

In this study, we designed a long amplicon panel to sequence *P*. *falciparum* drug resistance related genes. It covers current artemisinin resistance associated targets (including *Pfk13* and three newly identified genes: *Pfcoronin*, *Pfubp1* and *Pfap2μ*) and common ACTs partner drugs resistance targets (*Pfmdr1* and *Pfcrt*). We also designed full-length gene constructs for *Pfk13*, *Pfcoronin*, *Pfap2μ* and *Pfcrt* to systematically investigate mutational profiles across entire coding regions and their correlations with antimalarial drug resistance. The method shows excellent detection performance and has strong practical application potential.

## Methods

### Designing multiplex amplicon panel

Through in silico optimization using *multiply* software [31], we designed specific primers for six targets with amplicon sizes standardized to approximately 2.5 ± 0.2 kb to minimize amplification bias. Full-length coverage was achieved for *Pfk13*, *Pfcoronin*, and *Pfap2μ*, while *Pfmdr1*, *Pfcrt* and *Pfubp1* were designed with partial sequences covering all known resistance-associated loci (Table 1). The *Pfmdr1* gene was strategically divided into two fragments in the experimental design.

**Table 1:** Target genes for amplicon sequencing panel

No	Reference ID	Gene name	CDS^a^	Amplicon^b^	Amino acids covered	Relevance
1	PF3D7_1343700	*pfkelch13*	2180	2583	1–726	Artemisinin
2	PF3D7_1251200	*Pfcoronin*	2144	2439	1–602	Artemisinin
3	PF3D7_0104300	*Pfubp1*	10,961	2302	1123–1876	Artemisinin
4	PF3D7_1218300	*Pfap2μ*	1865	2462	1–621	Artemisinin
5	PF3D7_0709000	*Pfcrt*	3095	2481	37–381	Chloroquine Piperaquine Amodiaquine Lumefantrine
6	PF3D7_0523000	*Pfmdr1*	4259	4819	1–418, 679–1419	Lumefantrine Pyronaridine Mefloquine

^a^CDS refers to the length of coding sequence. ^b^Amplicon refers to the length of amplicon sequence

### Construction of the mock samples

We cultured the *P*. *falciparum* 3D7 strain to reach a parasite density of 2% (50,000 parasites/μL). The infected blood was then mixed with uninfected blood in various ratios to generate samples mimicking parasitemia levels of 1%, 0.1%, 0.01%, 0.005%, 0.001%, and 0.0001%. Following this, 150 μL of each mixture was spotted onto filter paper and air-dried under ambient conditions to generate dried blood samples (DBS). Genomic DNA was extracted using a QIAamp DNA Mini Kit (QIAGEN; Düsseldorf, Germany).

### Collection of field clinical samples

Venous blood samples were collected from 16 returned migrant workers originating from Democratic Republic of Congo in Jiangsu Province from January 2020 to December 2023. *Plasmodium* species were identified through microscopic examination, rapid diagnostic test (RDT), and real time fluorescence quantitative PCR (qPCR). All samples were confirmed for parasite density via qPCR [32]. The study was approved by the Ethics Committee of Jiangsu Institute of Parasitic Diseases (approval number JIPD-2024–017) and written informed consent was obtained from each participant and parent or legal guardian.

### Laboratory protocol and sequencing

Primer concentration combinations were evaluated utilizing gel electrophoresis and sequencing. The optimization process was designed to identify robust primer sets that met two critical performance criteria: the ability to achieve detection thresholds of ≤ 5 parasites/μL in both DBS and VB samples during successful amplification, and the minimization of nonspecific banding. After multiple rounds of multiplex PCR optimization, the final reaction conditions used in this study were determined, ensuring experimental precision and reproducibility. In brief, 4 μL of gDNA from VB and DBS mock samples were used as template in a 20 μL multiplex PCR with UCP Multiplex PCR kit (#206,472) and amplicon panels pools (Table S1 and S2). Multiplex PCR products were cleaned using a 0.6 × ratio of QIAseq Beads (#333,923) and eluted in 25 μL nuclease-free water. Amplicon quality was evaluated using the 1 × dsDNA High Sensitivity Assay on a Qubit Fluorometer (Invitrogen) before library preparation. The remaining PCR products were purified for paired-end sequencing (2 × 150 bp chemistry) of the final pool was performed on the Illumina NovaSeq 6000 platform and using the VAHTS Universal Pro DNA Library Prep Kit (Illumina).

### Bioinformatics analysis

Illumina raw paired-end reads were quality-controlled and filtered using fastp [33]. Following this step, reads that are mapped to the human reference genome were discarded. The remaining reads were mapped to the *P*. *falciparum* 3D7 v3 reference genome [34] using BWA-MEM 0.7.17 [35] with the -M parameter to mark shorter split hits as secondary. BAM improvement steps were applied to the read mapping outputs. Samtools fixmate v1.20 [36] and Picard v3.2.0 MarkDuplicates were applied to the BAM files of each sample. GATK Base Quality Score Recalibration [37] was applied using default parameters, using variants from the *P*. *falciparum* crosses 1.0 release [34] as known sites. Genotypes were called from each sample-level BAM using GATK HaplotypeCaller with parameters -contamination 0 -ERC GVCF to produce a separate gVCF. Each variant was assigned a quality score using GATK’s Variant Quality Score Recalibration (VQSR). VariantRecalibrator was run using the PASS variants from the *P*. *falciparum* crosses 1.0 release as a training set. ApplyRecalibration was then used to assign each variant a quality score named VQSLOD. Variants annotations were applied using snpEff version 5.2c [38], with gene annotations downloaded from GeneDB6 February 2020 release [34].

## Results

### Design of the amplicon panel and amplification performance evaluation

**Fig. 1:**
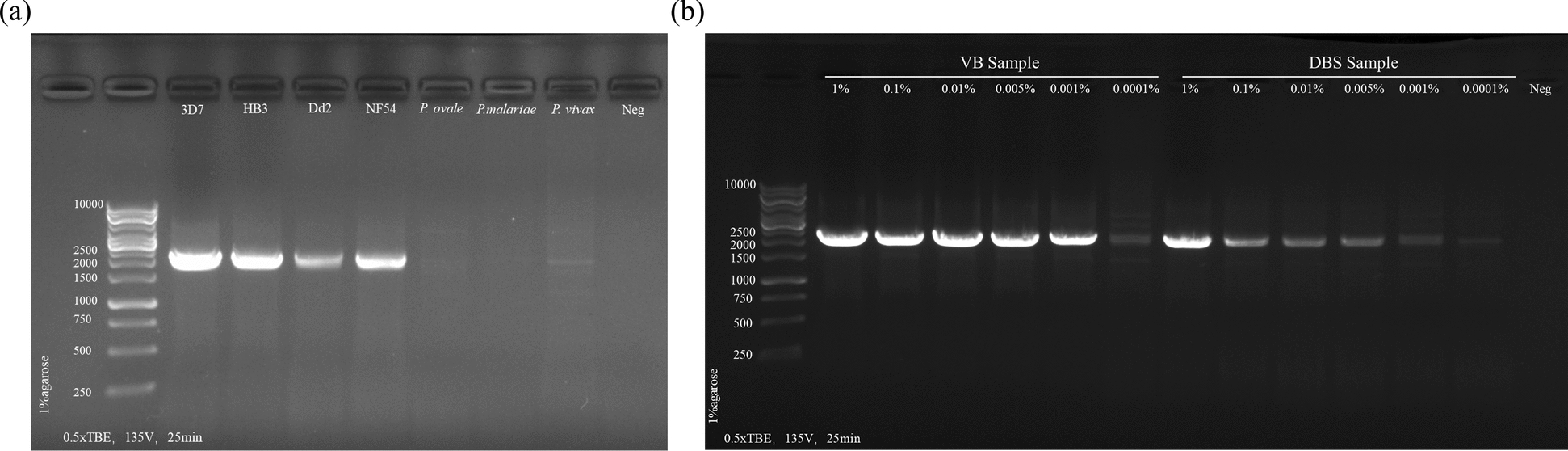
The amplification effect of the method. (**a**) Gel electrophoresis of amplified products of different strains of *P*. *falciparum* and *Plasmodium* species. (**b**) Gel electrophoresis of amplification products of mock samples with different parasitemia (1%-0.0001%)

We developed a long-amplicon panel (Table 1) encompassing four artemisinin resistance-related genes (*Pfk13*, *Pfcoronin*, *Pfap2μ* and *Pfubp1*) and two partner drug resistance-related genes (*Pfmdr1* and *Pfcrt*), all primer sequences detailed in Table S1. Through iterative optimization via gel electrophoresis and high-throughput sequencing, we determine the optimal annealing temperatures and establish a tiered primer concentration system: *Pfk13* at 2.5 μM, other targets at 0.5 μM (Fig. S1a and S1b). Specificity validation involved six *Plasmodium* strains: four *P*. *falciparum* (3D7, Dd2, HB3 and NF54) and three non-falciparum species (*P.ovale, P.vivax* and *P.malariae*). Gel electrophoresis demonstrated distinct amplification bands (2.5 ± 0.2 kb) in all *falciparum* strains, with no specific amplification in nontarget species (Fig. 1a). Deep sequencing revealed 100% target gene coverage in *falciparum* samples versus < 3.7% nonspecific mapping in other species, confirming primer specificity across the *Plasmodium falciparum* genus (Fig. S2c). The impact of varying parasitemia on amplification efficiency was also evaluated (Fig. 1b). For DBS and VB samples, gel electrophoresis showed distinct amplification bands (2.5 ± 0.2 kb) range for samples with 1% to 0.001% parasitemia, but no nonspecific bands. However, samples with 0.0001% parasitemia had significantly weaker band intensity (Fig. 1b).

### Sequencing performance of mock and field samples

**Fig. 2:**
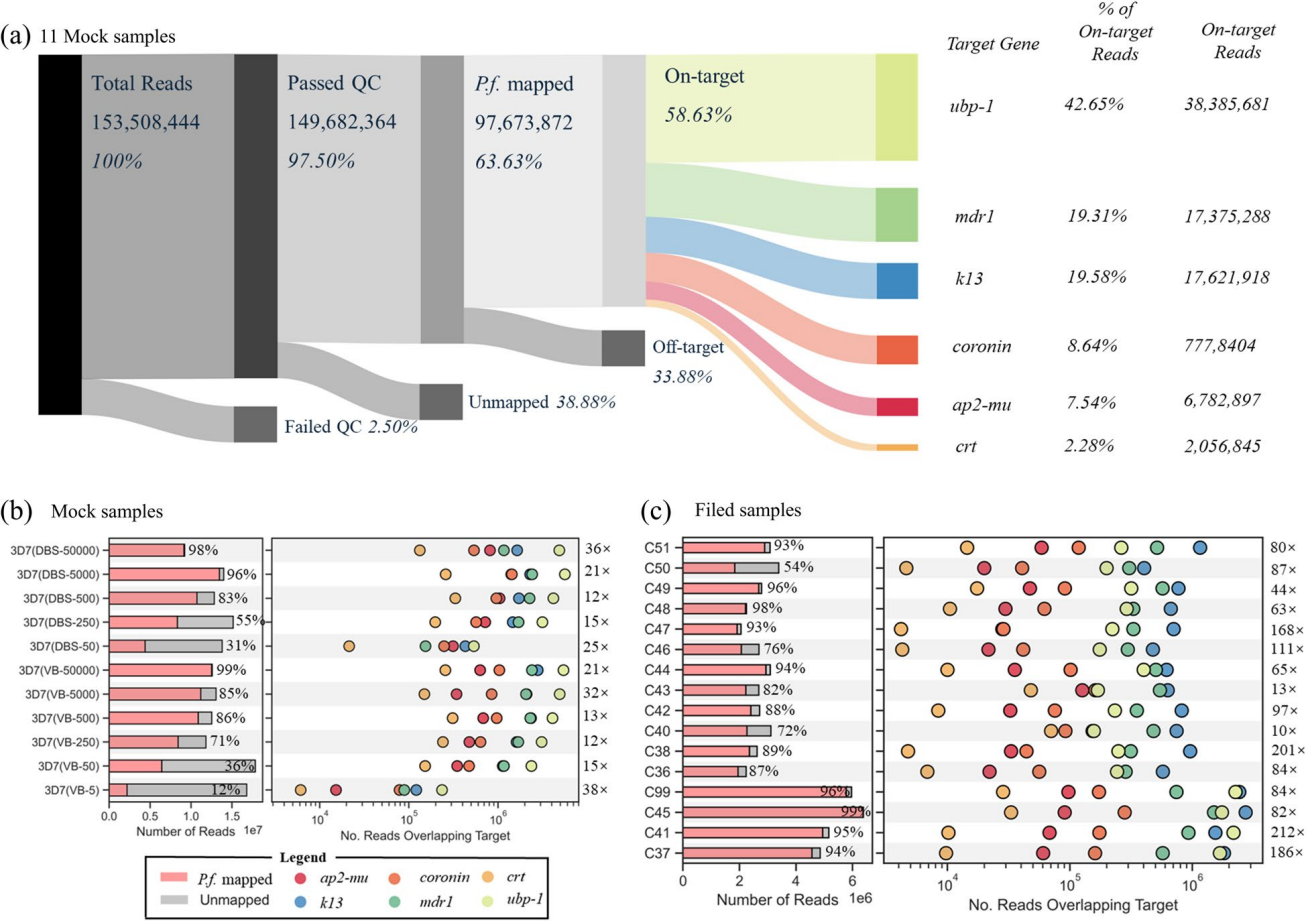
Sequencing coverage across samples and target genes for the long-amplicon panel. (**a**) Reads of mock samples generated from Illumina Novaseq sequencing. The upper right corner shows the total sequencing reads (153,508,444; 100%), which are subsequently filtered during the data analysis process to yield the reads of interest—specifically those mapped to target genes (90,002,000; 58.63%). (**b**) The left panel presents a bar plot showing the total number of reads generated for each sample, stratified by mapping outcome reads mapped to *P*. *falciparum* (*P.f.*) (red) and those failing to map (grey). The right panel features a scatter plot illustrating the number of reads that overlap each target gene (color-coded) after mapping for each sample. (**c**) Same as (**b**) for 16 field samples collected as VB from the Democratic Republic of Congo

A total of 11 mock samples were used to validate the efficacy of multi-target amplification sequencing. The total raw sequencing data generated from 11 mock samples using the Illumina platform comprised 153,508,444 reads, equivalent to 21.43 Gb of sequence data (Fig. 2a). Of these, 97.50% were retained after processing with the fastp quality-control filter. Most reads successfully mapped to the reference sequence (97,673,872 reads; 63.63%, Fig. 2a). Among the reads successfully aligned to the *P*. *falciparum* genome, 92.14% were mapped to the intended target regions, indicating method effectively reduced off-target amplicon generation. Finally, 56.63% of all sequencing reads were mapped to on-target regions.

We evaluated sequencing coverage evenness across amplicons by quantifying overlapping reads for each target. For DBS mock samples, VB mock samples, and field samples, the median fold-difference in coverage between the highest and lowest abundant amplicons was 21, 18, and 84, respectively (Fig. S3a and S3c). For both mock and field sample sequencing runs, the amplicon abundance rank order showed no statistical differences (Spearman’s ρ = 0.81 for mock samples; Spearman’s ρ = 0.71 for field samples; Fig. S3a and S3b). Furthermore, no significant difference was found between two groups (Spearman’s ρ = 0.771, MedianΔ = 0; Fig. S4), while *Pfk13* ranked first in field samples but third in mock samples, and *Pfubp1* showed the opposite. *Pfcrt* had the lowest abundance in both mock and field samples. However, *Pfcrt* still had a median coverage of 197,673-fold in mock samples and 10,081-fold in field samples (Fig. S3a and S3b).

### The impact of parasitemia on sequencing performance

**Fig. 3:**
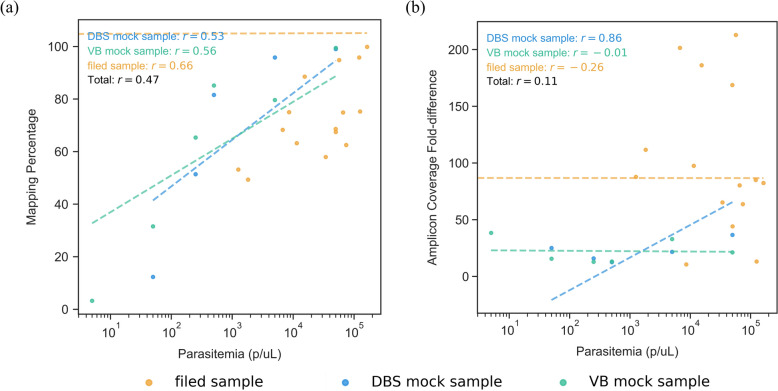
Effect of parasitemia on sequencing performance. Scatterplots display the effect that parasitemia (x-axis) has on the long-amplicon panel. (**a**) “Mapping percentage,” which is the percentage of all reads from a sample that mapped to *P*. *falciparum*. (**b**) The “amplicon coverage fold-difference” which, for a given sample, is the ratio of the number of reads overlapping the highest abundance amplicon, divided by the number of reads overlapping the lowest abundance amplicon. Each point is either a DBS mock sample (blue), or a field sample (yellow) or a VB mock sample (green)

We investigated the impact of varying parasitemia density on sequencing performance metrics: the percentage of reads mapped to *Plasmodium falciparum*; and the fold difference in coverage between the most abundant and least abundant amplicons. (Fig. 3). *P*. *falciparum* mapping percentages showed a moderate positive correlation with parasitemia (Pearson’s *r* = 0.47; Fig. 3a). However, the amplicon coverage fold-difference had only a weak positive correlation with parasitemia (Pearson’s *r* = 0.11; Fig. 3b).

**Fig. 4:**
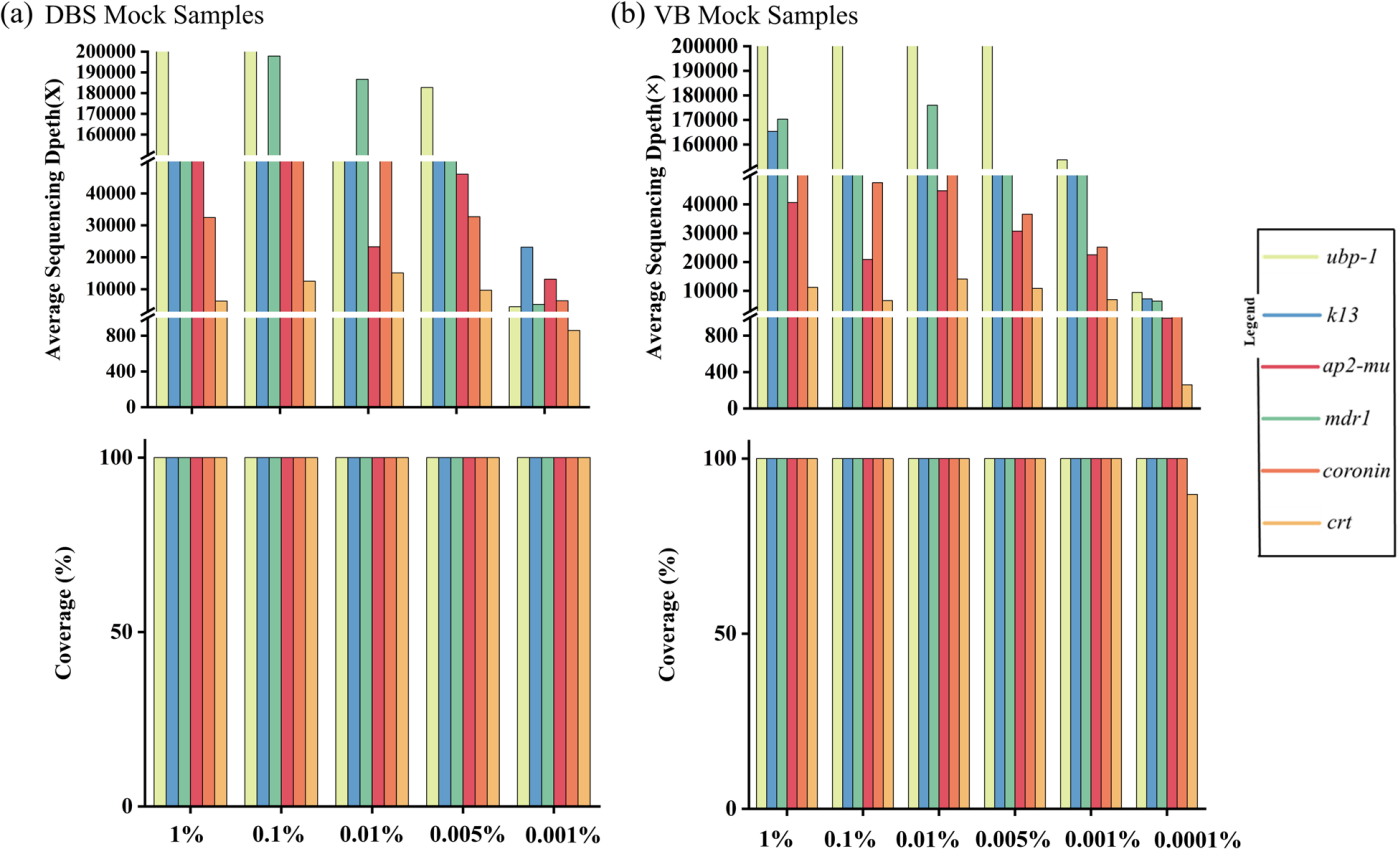
The sensitivity of multiple PCR amplicon sequencing. Two measures of sequencing analysis are shown (y-axis): The average sequencing depth of the target regions; The coverage of sequencing depth > 100 × . (**a**) Bar plot the average sequencing depth and coverage of target genes for five DBS samples (parasitemia ranging from 1% to 0.001%). (**b**) Same as (**a**) but for 6 VB mock samples (parasitemia ranging from 1% to 0.0001%) sequenced

We evaluated the sensitivity of our protocol using amplicon panels on mock samples. Figure 4 shows that as parasite density decreases, the average sequencing depth of target regions declines (Fig. 4b), yet the depth with > 100 × coverage stays at 100%. Notably, for the DBS sample with 0.001% parasitemia, the average sequencing depth of the target regions was > 858 × . For VB mock samples, the average sequencing depth showed a similar trend to that of DBS mock samples (Fig. 4b). Remarkably, even at 0.0001% parasitemia, the sample’s target regions had an average sequencing depth of > 1346 × , but the coverage of the *Pfcrt* gene region at > 100 × was 89.77% (Fig. 4b).

### Assessment of the minimum sequencing data volume

**Fig. 5:**
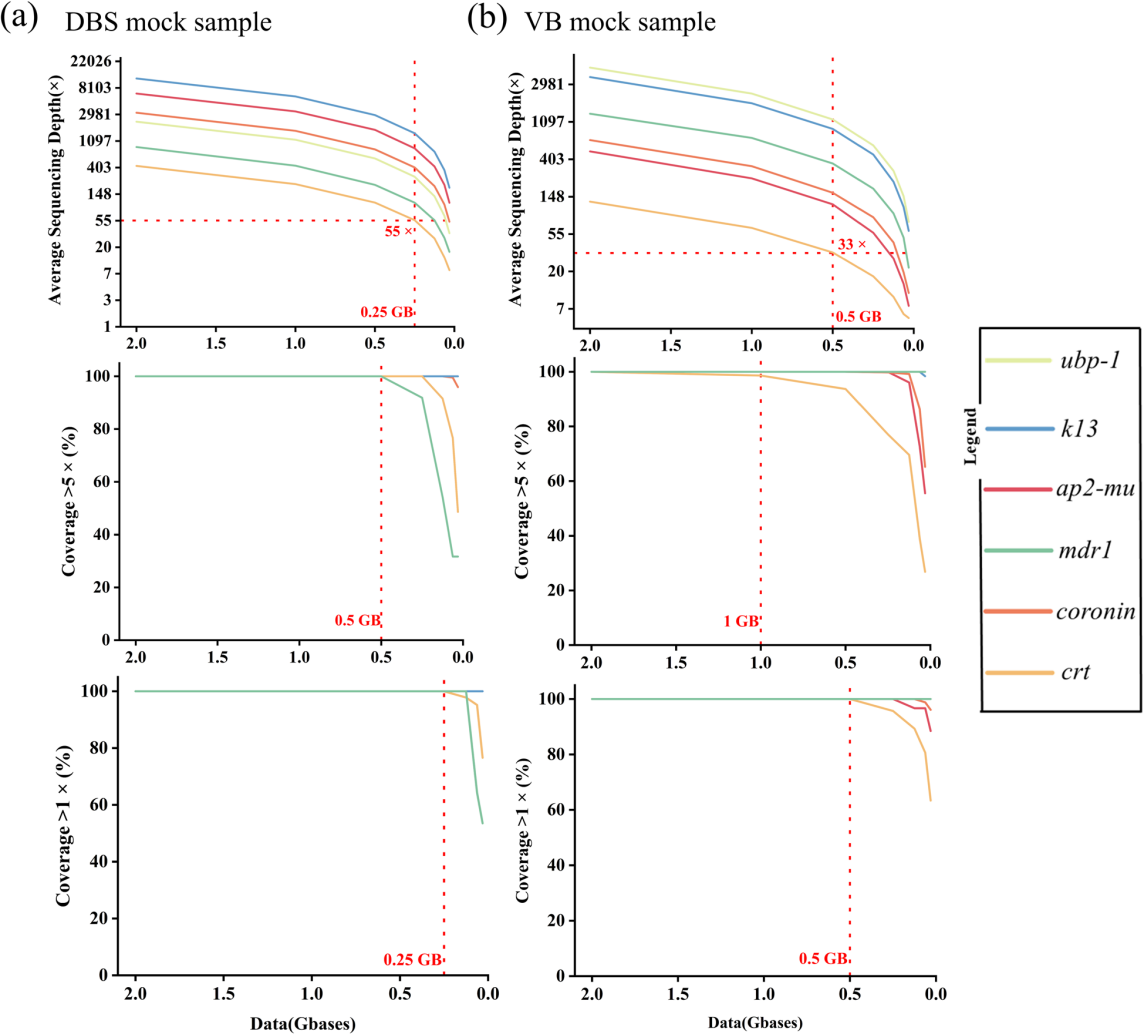
Exploration of the minimum sequencing data volume for sequencing mock samples. Three measures of sequencing performance are shown (y-axis): “average sequencing depth”, which is the average sequencing depth of target genes (top row); “coverage > 5 × (%)”, which is the percentage of sequencing depth greater than 5 × (middle row); “coverage > 1 × (%)”, which is the percentage of a sequencing depth greater than 1 × (bottom row). (**a**) The relationship between three measures of DBS mock sample with 0.001% parasitemia and changes in data volume. (**b**) Same as (**a**), the sample is VB mock sample with 0.0001% parasitemia

To meet minimal sequencing data requirements, we used *fastp* software to iteratively trim raw data and evaluated the sequencing efficacy post-reduction. Sensitivity analysis revealed a negative correlation between sequencing efficiency and parasitemia at a fixed data volume. Consequently, we concentrated optimization efforts on VB mock samples (0.0001% parasitemia) and DBS mock samples (0.001% parasitemia). The total cost for processing a sample from DNA extraction to the acquisition of sequencing data is as low as $15.60 (Table S3). For DBS mock samples, reducing data volume decreased the average target region sequencing depth (Fig. 5a). The percentage of target regions with sequencing depths exceeding 5 × remained 100% until the data volume fell below 0.50Gb. However, some target genes had a coverage rate of less than 100% when data volume was below 0.25GB. At 0.25GB, the minimum average sequencing depth of the target gene reached 55 × . For VB samples with 0.0001% parasitemia, the average target region sequencing depth showed a similar trend to DBS samples as data volume decreased (Fig. 5b). A threshold was observed at 1.0Gb: below this, some target regions had less than 100% of sequencing depths exceeding 5 × . Notably, when data volume was below 0.5 GB, target region coverage was less than 100%, and the minimum average sequencing depth of the target gene reached 33 × .

## Discussion

In this study, we designed a long-amplicon panel for antimalarial resistance molecular surveillance which combines full-length sequencing of artemisinin resistance-associated genes (*Pfk13*, *Pfcoronin*, *Pfap2μ*) with partial sequencing of loci linked to partner drug resistance (*Pfmdr1*, *Pfcrt*) and artemisinin resistance (*Pfubp1*). In contrast to prior methodologies restricted to hotspot regions [28], our approach encompasses the entirety of coding sequences, enabling the identification of both known and novel mutations. Moreover, comparative analysis (Table S4) shows that our method offers measurable gains in both specificity and sensitivity over other published approaches [29–31, 39, 40], while keeping the per-sample cost to $15.60. This represented a 290% cost reduction from the $60.87 per-sample expenditure reported in Chanon Kunasol et al.’s protocol [41] and 60% lower than the $25.00 per-sample baseline established by Mariateresa de Cesare et al. [31].

Current ART-R monitoring mainly detects the *Pfk13* domain region (441–720) [42, 43] but ignores nondomain mutations [44]. While current MIPs and nanopore sequencing panels are effective for predefined resistance loci genotyping [28], they do not currently cover the entirety of emerging genes, such as *Pfcoronin*, *Pfubp1* and *Pfap2μ*, which have recently been implicated in artemisinin resistance, leaving novel mutations undetected. Our long-amplicon panel covers the entire *Pfk13* and three additional artemisinin resistance-related genes. The iterative optimization of primer concentrations and annealing temperatures ensured minimal amplification bias, even at ultralow parasitemia. The Gel electrophoresis and sequencing validation confirmed robust amplification specificity across *P*. *falciparum* strains, with less than 3.7% nonspecific mapping in non-falciparum species, demonstrating specificity critical for field applications.

The panel demonstrates exceptional sensitivity, with detection thresholds as low as 0.001% (DBS) and 0.0001% (VB) parasitemia for resistance markers, exceeding standard surveillance detection limits in malaria-endemic regions. Furthermore, this high level of sensitivity ​strongly supports the assay’s reliability in identifying minority alleles​ within complex polyclonal infections. Significantly, the absence of statistical differences in the rank order of amplicon abundance between mock and field samples indicates that PCR efficiency variations among targets are consistent and can be effectively controlled via standardized amplification protocols. Comparison of amplicon abundance rankings between mock and field samples revealed high concordance, demonstrating that laboratory-prepared mock samples can effectively replicate real-world sampling conditions. Differences in amplicon abundance rankings between mock and field samples likely reflect natural infection backgrounds such as variable parasite densities, polyclonal infections, and genomic sequence variation. A study also observed this phenomenon, indicating sample-set dependent effects [31]. Specifically, *Pfcrt* remained the lowest abundance for both mock and field samples, attributable to its hyper-AT-rich sequence (80.33%, A + T) and long homopolymers. This highlights the importance of validating surveillance tools across diverse field backgrounds to account for genetic architecture.

The long-amplicon panel demonstrates that the robust performance in low-parasitemia DBS samples is remarkable. DBS-based surveillance is logistically advantageous in resource-limited settings [45, 46], however, existing methods often struggle to yield adequate sequencing data due to low DNA extraction efficiency. Our protocol achieved > 100 × coverage for all targets in VB sample at 0.001% parasitemia, proving feasible for cost-effective, high-throughput applications. The slight decline in *Pfcrt* coverage at 0.0001% parasitemia in VB mock samples primarily stems from the combined effects of its hyper-AT-rich sequence (80.33%, A + T), exceptionally long amplicon length (2481 bp), long homopolymer and very low parasitemia, all of which significantly reduce amplification efficiency [31, 39]; future iterations could address this by refining primer designs or adding spike-in controls for low-abundance targets.

The inclusion of *Pfcoronin*, *Pfubp1*, and *Pfap2μ* genes are associated with ART-R in African [47, 48] and imported cases [21].This establishes our panel as a vital tool for monitoring resistance evolution beyond the *Pfk13* locus. Detecting mutations in these genes, even without *Pfk13* variants, implies that relying solely on *Pfk13* may underestimate ART-R prevalence in certain settings. Furthermore, the integrating *Pfmdr1* and *Pfcrt* enables simultaneous partner-drug-resistance surveillance, which is critical for evaluating ACT efficacy as lumefantrine and piperaquine resistance spreads [49]. By capturing haplotype diversity across full-length genes, the panel offers insights into resistance mechanisms and potential compensatory mutations, which are essential for predicting resistance trajectories.

Although the long-amplicon methodology represents a significant technical advancement, some limitations still need consideration. First, the current design focuses only on six genes and adding more resistance loci (e.g., *Pfatp6* for artemisinin [50] or *Pfpm2/3* for piperaquine [51]) could further enhance its utility. Second, the filed sample (*n* = 16) only included Democratic Republic of Congo migrant workers. Larger studies across different geographic areas are needed to assess its performance in various transmission settings. Third, as it relies on Illumina sequencing, its real-time use in the field is limited. Future integration with portable platforms like nanopore sequencing might allow decentralized, near-real-time resistance monitoring.

## Conclusions

This study demonstrates that an optimized long-amplicon sequencing panel can highly sensitively, specifically and comprehensively detect antimalarial resistance markers. Our method addresses limitations in existing surveillance tools by offering a scalable solution to monitor the evolution of resistance and inform treatment policy decisions. As resistance spreads, integrating these panels into routine surveillance will be critical for sustaining global malaria control achievements.

## Supplementary Information

Supplementary file 1. Table S1. Sequence of all amplicon primers. Table S2. PCR protocol. Table S3. Consumable cost based on multiplex targeted amplicon sequencing. Table S4. Comparison of performance of different panels. Fig. S1. Amplified product gel electrophoresis. Fig. S2. Sequencing quality of different *plasmodium*. Fig. S3. Order of amplicon abundances for the long-amplicon panel in mock and field samples. Fig. S4. Difference test between mock samples and filed samples

## Data Availability

Data supporting the main conclusions of this study are included in the manuscript.

## Abbreviations

ACTs: Artemisinin-based combination therapies
VB: Venous blood
DBS: Dried blood spots
WHO: The World Health Organization
AL: Artemether-lumefantrine
AS-AQ: Artesunate-amodiaquine
AS-MQ: Artesunate methylfluoroquine
DHA-PPQ: Dihydroartemisinin-piperaquine
GMS: The Greater Mekong Subregion
*Plasmodium falciparum*: *P*. *falciparum*
*Pfcoronin*: *P*. *falciparum* Coronin
*Pfubp1*: *P*. *falciparum* Ubiquitin carboxyl-terminal hydrolase 1
*Pfap2μ* (*Pfap2-mu*): *P*. *falciparum* AP-2 complex subunit mu
*Pfmdr1*: *P*. *falciparum* Multidrug resistance protein 1
*Pfcrt*: *P*. *falciparum* Chloroquine resistance transporter
MIPs: Molecular inversion probes
RDT: Rapid diagnostic test
qPCR: Real time fluorescence quantitative PCR
