# Supplementary material for: Multiplex long-amplicon sequencing for comprehensive molecular surveillance of Plasmodium falciparum resistance to artemisinin and partner drugs in artemisinin-based combination therapies (ACTs)
Source: Parasit Vectors. 2025 Oct 21;18:419. doi: 10.1186/s13071-025-07058-6 (PMC12539163; doi:10.1186/s13071-025-07058-6)
Supplement: Supplementary file 1 — Table S1. Sequence of all amplicon primers. Table S2. PCR protocol. Table S3. Consumable cost based on multiplex targeted amplicon sequencing. Table S4. Comparison of performance of different panels. Fig. S1. Amplified product gel electrophoresis. Fig. S2. Sequencing quality of different plasmodium. Fig. S3. Order of amplicon abundances for the long-amplicon panel in mock and field samples. Fig. S4. Difference test between mock samples and filed samples [file 13071_2025_7058_MOESM1_ESM.docx]

**Supplementary Material**

**Table S1.** Sequence of all amplicon primers.

| Target | Primer^a^ | Direction | Sequence | Start Position | End Position |
| --- | --- | --- | --- | --- | --- |
| *Pfap2μ* | AP2-mu_v7_F | F | AGCGTCATTTGTTCTTCCGTTTT | 717912 | 717934 |
| *Pfap2μ* | AP2-mu_v7_R | R | ACAACGTATAAAGGAAACGGAAATTCT | 720347 | 720373 |
| *Pfcoronin* | Coronin_v8_F | F | TTCTTTGTCTACTGCGGACTATT | 2091814 | 2091836 |
| *Pfcoronin* | Coronin_v8_R | R | CTGATGTGCCCCTCCCCC | 2094235 | 2094252 |
| *Pfk13* | K13_v0_F | F | AGGGAAAATCATAAACAATCAAGTAA | 1724568 | 1724593 |
| *Pfk13* | K13_v0_R | R | ACAAGGCGTAAATATTCGTGT | 1727130 | 1727150 |
| *Pfubp1* | Ubp1part_v8_F | F | AGCGCGTTCTTCATTCCAAAA | 193614 | 193634 |
| *Pfubp1* | Ubp1part_v8_R | R | GCATACATATTGCTGTCCTCCC | 195894 | 195915 |
| *Pfcrt* | crtpart_v4_F | F | GATGGCTCACGTTTAGGTGG | 403488 | 403507 |
| *Pfcrt* | crtpart_v4_R | R | ATGAACGAACAAGCCATTTGAT | 405947 | 405968 |
| *Pfmdr1* | mdr1part1_v9_F | F | GCCTTTATCTATATTACATTTTGCACC | 956683 | 956709 |
| *Pfmdr1* | mdr1part1_v9_R | R | TCCCACAACCTGATTCTCCC | 959124 | 959143 |
| *Pfmdr1* | mdr1part2_v3_F | F | TGAACAAGGTACACATGATAGTCT | 959901 | 959924 |
| *Pfmdr1* | mdr1part2_v3_R | R | ACGGACAAGAGTTGATACTGTTC | 952236 | 962258 |

^a^ For multiplex PCR primer preparation, *Pfk13* primers (K13_v0_F/R) were used at 100 μM working concentration, while all other primers were maintained at 10 μM standard concentration.

**Table S2.** PCR protocol.

| Step | Temperature (°C) | Time | Cycles |
| --- | --- | --- | --- |
| Initial denaturation | 95 | 10min | 1 |
| Denaturation | 95 | 15s | 35 |
| Annealing | 55 | 15s |  |
| Extension | 60 | 6min |  |
| Final Extension | 60 | 10min | 1 |
| Hold | 4 | - |  |

**Table S3.** Consumable cost based on multiplex targeted amplicon sequencing

| Step | Item | Supplier | Item Code | Cost per Sample (USD) |
| --- | --- | --- | --- | --- |
| DNA Extraction | QIAamp DNA Mini QIAcube kit(240) | Qiagen | 51326 | 3.87 |
| Multiplex PCR | Primers pool |  |  | 0.03 |
| Multiplex PCR | UCP Multiplex PCR kit(500) | Qiagen | 206744 | 1.94 |
| purifying | QIAseq Beads(55mL) | Qiagen | 333903 | 0.08 |
| Library construction and sequencing |  |  |  | 9.68 |
|  |  |  | Total | 15.6 |

Table S4. Comparison of performance of different panels.

| Panel | Specificity | Senstive^a^ | Cost per Sample (USD) | Reference |
| --- | --- | --- | --- | --- |
| This study | Species-Specific | VB^b^: 5 parasite/μL  DBS^b^: 50 parasite/μL | 15.60 | - |
| MAD^4^HatTeR | - | DBS:100 parasite/μL | 12-25 | [30] |
| NOMADS8/S16 | - | VB:1000 parasite/μL | 25 | [31] |
| - | - | VB and DBS: 100 parasite/μL | 35 | [29] |
| SpotMalaria | - | VB: 50 parasite/μL | - | [40] |
| AmpliSeq | - | DBS: 60 parasite/μL | - | [39] |
| TADs | - | - | 60.87 | [41] |

^a^Sensitive means the minimum parasitaemia that can be accurately detected. ^b^ VB and DBS refer to VB samples and DBS samples, respectively. -^c^ means the assessment was not conducted.

**Fig. S1.** Amplified product gel electrophoresis. (a) Gel electrophoresis of amplification products at different annealing temperatures. DBS mock samples with a parasite density of 1 % were used for amplification. (b) Gel electrophoresis of amplification products in different primer concentration systems. The primer concentration systems from left to right are as follows: the primer of *pfk13* is 2.5 μM and the primer of other genes are 1.5 μM; the primer of *pfk13* are 2.5 μM and the primer of other genes are 1.0 μM; the primer of *pfk13* is 2.5 μM and the primer of other genes are 0.5 μM; the primer of *pfk13* is 2.5 μM and the primer of other genes are 0.25 μM; the primer of *pfk13* is 5.0 μM and the primer of other genes are 1.5 μM; the primer of *pfk13* is 5.0 μM and the primer of other genes are 1.0 μM; the primer of *pfk13* is 2.5 μM and the primer of other genes are 0.5 μM; the primer of *pfk13* is 2.5 μM and the primer of other genes are 0.25 μM. 0.1% parasitaemia DBS mock sample was used as an amplification template DNA.


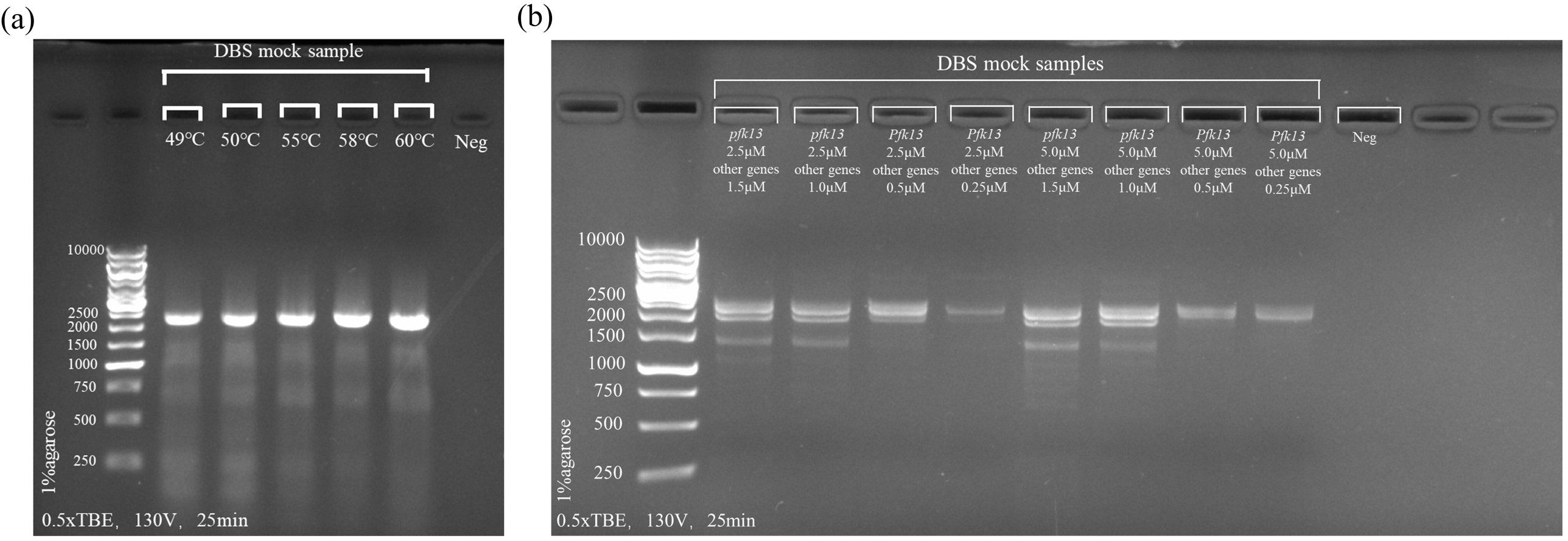


**Fig. S2.** Sequencing quality of different *plasmodium*. (a) The total number of reads from sequencing different *plasmodium* samples and the number of reads mapped to the target regions. (b) The proportion of reads mapped to the target regions from sequencing different *plasmodium* samples. (c) The coverage of the target region's depth from sequencing different *plasmodium* samples. (d) The average sequencing depth of target regions from sequencing different *plasmodium* samples.





**Fig. S3.** Order of amplicon abundances for the long-amplicon panel in mock and field samples. (a) Long-amplicon panel data for 11 mock samples. The left panel presents a scatter plot showing the amplicon abundances (x-axis) for each target (y-axis). The median number of reads per amplicon is indicated in brackets next to each amplicon target name. The right panel features a heatmap with amplicons as rows and their rank order by abundance as columns. Spearman’s ρ for amplicon abundances across samples is displayed above the heatmap. Amplicons are ordered on y-axis by their median ranking. (b) Same as (a) but for 16 field samples sequenced.


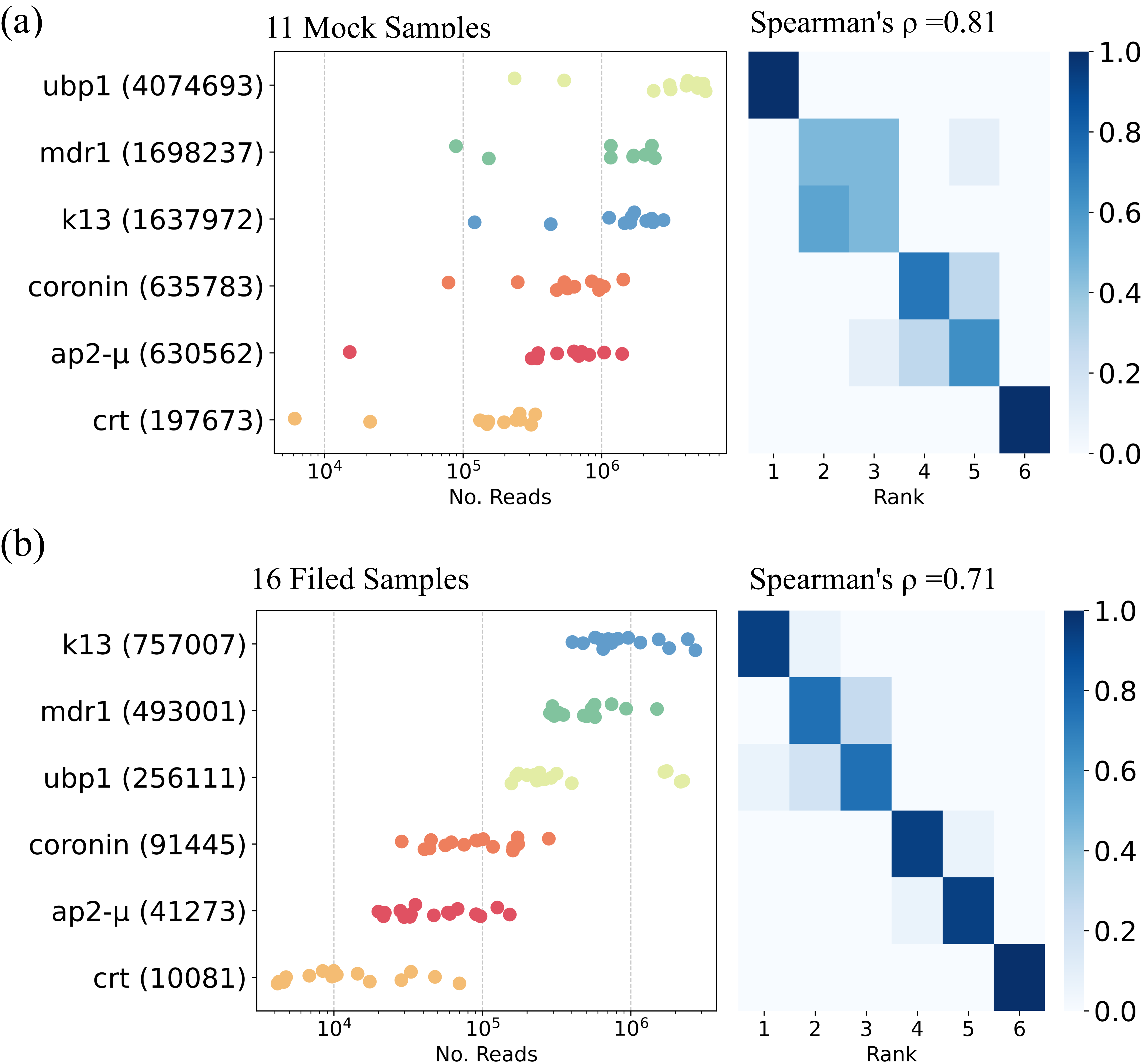


**Fig. S4.** Difference test between mock samples and filed samples. (a) Left: Scatter plot comparing amplicon abundance ranks (1 = most abundant) in mock vs. field samples. Dashed diagonal line (y = x) represents perfect rank agreement. Spearman's correlation coefficient (ρ) with permutation-based p-value (two-sided, n = 10,000 resamples) is shown in the inset; (b) Right: Distribution of rank differences (Field rank - Mock rank). Positive values indicate lower abundance ranks in field samples. Red dashed line marks the median difference across all amplicons. Histogram bins represent integer differences in ranking positions.

**

**
